# Feasibility test of a UK-scalable electronic system for regular collection of patient-reported outcome measures and linkage with clinical cancer registry data: The electronic Patient-reported Outcomes from Cancer Survivors (ePOCS) system

**DOI:** 10.1186/1472-6947-11-66

**Published:** 2011-10-26

**Authors:** Laura Ashley, Helen Jones, David Forman, Alex Newsham, Julia Brown, Amy Downing, Galina Velikova, Penny Wright

**Affiliations:** 1 Applied Informatics and Cancer Care research team, Psychosocial Oncology and Clinical Practice Research Group, University of Leeds; based at St James's Institute of Oncology, Beckett Street, Leeds, LS9 7TF, UK; 2 International Agency for Research on Cancer, 150 Cours Albert Thomas, 69372 Lyon CEDEX 08, France; 3 Psychosocial Oncology and Clinical Practice Research Group, University of Leeds; based at St James's Institute of Oncology, Beckett Street, Leeds, LS9 7TF, UK; 4 Clinical Trials Research Unit, University of Leeds, Leeds, LS2 9JT, UK; 5 Northern and Yorkshire Cancer Registry and Information Service, St James's Institute of Oncology, Beckett Street, Leeds, LS9 7TF, UK

## Abstract

**Background:**

Cancer survivors can face significant physical and psychosocial challenges; there is a need to identify and predict which survivors experience what sorts of difficulties. As highlighted in the UK National Cancer Survivorship Initiative, routine post-diagnostic collection of patient reported outcome measures (PROMs) is required; to be most informative, PROMs must be linked and analysed with patients' diagnostic and treatment information. We have designed and built a potentially cost-efficient UK-scalable electronic system for collecting PROMs via the internet, at regular post-diagnostic time-points, for linking these data with patients' clinical data in cancer registries, and for electronically managing the associated patient monitoring and communications; the electronic Patient-reported Outcomes from Cancer Survivors (ePOCS) system. This study aims to test the feasibility of the ePOCS system, by running it for 2 years in two Yorkshire NHS Trusts, and using the Northern and Yorkshire Cancer Registry and Information Service.

**Methods/Design:**

Non-metastatic breast, colorectal and prostate cancer patients (largest survivor groups), within 6 months post-diagnosis, will be recruited from hospitals in the Yorkshire Cancer Network. Participants will be asked to complete PROMS, assessing a range of health-related quality-of-life outcomes, at three time-points up to 15 months post-diagnosis, and subsequently to provide opinion on the ePOCS system via a feedback questionnaire. Feasibility will be examined primarily in terms of patient recruitment and retention rates, the representativeness of participating patients, the quantity and quality of collected PROMs data, patients' feedback, the success and reliability of the underpinning informatics, and the system running costs. If sufficient data are generated during system testing, these will be analysed to assess the health-related quality-of-life outcomes reported by patients, and to explore if and how they relate to disease, treatment and/or individual differences characteristics.

**Discussion:**

There is currently no system in the UK for collecting PROMs online and linking these with patients' clinical data in cancer registries. If feasible, ePOCS has potential to provide an affordable UK-scalable technical platform to facilitate and support longitudinal cohort research, and improve understanding of cancer survivors' experiences. Comprehensive understanding of survivorship difficulties is vital to inform the development and provision of supportive services and interventions.

## Background

### Improving understanding of cancer survivors' experiences

The UK National Cancer Survivorship Initiative (NCSI) highlights a pressing need to improve understanding of cancer survivors' experiences and outcomes, through increased collection of patient reported outcome measures (PROMs, i.e. questionnaires) in the years post-diagnosis and treatment [1]. Research has highlighted that some cancer survivors experience persistent physical and psychosocial difficulties, yet has also shown that for many survivors quality-of-life is similar to the general population [2-7]; as studies to date have mostly examined limited short-term outcomes in small, non-UK samples, knowledge about who experiences what sorts of difficulties and when is limited [8,9]. Such knowledge is crucial, however, to inform the development and targeted provision of support services and interventions. As cancer survivors are a large and growing group, targeting support toward those most in need is increasingly important; although there are currently 2 million survivors in the UK, this is estimated to near double by 2030 [10]. To gain a comprehensive understanding of survivorship, PROMs need to be repeatedly collected from large numbers of patients, across multiple health-related quality-of-life domains for many years. To be of greatest use, PROMs also need to be linked and analysed with patients' diagnostic and treatment information, as this will enable identification of clinical predictors of survivorship difficulties, and thus facilitate early risk stratification.

### Overcoming the challenges of regularly collecting cancer PROMs and linking with patients' clinical data

A major challenge to large-scale, long-term PROMs collection is cost. The expense traditionally incurred using a paper-based methodology, however, can be reduced by over 75% through use of a largely electronic and/or internet-based system [11,12]; administering PROMs online, communicating with patients via email, and using a tracking database to semi-automate patient monitoring and management, avoids the expense of printing and postage and greatly reduces administrative staff costs. Another key challenge to successful PROMs collection is achieving high-levels of sustained patient participation [13,14]. However, online PROMs may also improve patient convenience, and thus participation and retention; internet-PROMs can be completed at any time of day, year-round, from patients' homes, as well as a growing number of web-accessible public locations and mobile devices.

Very little cancer survey research includes a means to link patients' questionnaires with their clinical data [15]; linking clinical data with other information can be logistically and ethically complex, time-consuming and expensive [16]. However, there is potential to mitigate the challenges of PROMs-clinical data linkage by using the UK's network of cancer registries which collate and store clinical data on all cancer patients [17]; English data are additionally pooled and stored by the National Cancer Intelligence Network in the National Cancer Data Repository (NCDR) [18]. Data linking via the registries/NCDR would capitalise on costly data collection and validation work already undertaken, and allow PROMs to be efficiently linked with a uniform dataset, which is subject to the highest standards of governance and security.

### Designing and developing an electronic system for regularly collecting PROMs online and linking with clinical cancer registry data

To facilitate and support increased and improved measurement of UK cancer survivors' experiences, we have designed and developed a technical *system *for regularly collecting PROMs online, at repeated post-diagnostic time-points, for linking and storing these with patients' clinical data in cancer registries, and for electronically managing the related patient monitoring and communications; the electronic Patient-reported Outcomes from Cancer Survivors (ePOCS) system [19]. The PROFILES Group (Patient Reported Outcomes Following Initial treatment and Long-term Evaluation of Survivorship) have very recently established a 'web-based registry' to facilitate cancer PROMs collection in The Netherlands, and which is linkable to clinical information in the regional Eindhoven cancer registry [20]. In the UK, however, there is currently no system for routinely collecting cancer PROMs and linking with patients' registry data.

In the ePOCS system, patients complete PROMs using a password-protected web-based questionnaire administration tool (QTool), which is accessed via a public-facing internet website (http://www.epocs.leeds.ac.uk), and the responses are imported and stored with patients' clinical data in the registries/NCDR. Patients click on PROMs listed on the QTool homepage, and move through the questions by clicking a 'Next Page', and lastly 'Submit', button. Patients can return to partially completed PROMs within 24 hours, and continue from where they left off; after this time, PROMs are automatically re-administered from the start. Submitted PROMs cannot be reviewed. Patients receive PROMs reminders if necessary, and thank you acknowledgements on completion of all measures. At new data collection time-points, patients receive invitations to go online again and complete further PROMs.

Prior to the start of data collection, ePOCS system administrators enter the required PROMs into QTool and specify, for each, for which patients each questionnaire is available (e.g. prostate cancer), from what time-point (e.g. 1 year post-diagnosis) and for how long (e.g. 3 weeks). During data collection, administrators track patients' PROMs activity and prepare and send related communications using a password-protected electronic tracking database (Tracker). The Tracker receives daily information on patients' PROMs completion progress from the QTool, and using this, automatically generates all necessary related correspondence (e.g. reminder notices) by populating pre-prepared communications with the relevant patient's details (e.g. name, address). Communications are generated, as appropriate, as ready-to-send emails or ready-to-print letters; ePOCS correspondence is entirely electronic for patients able and willing to provide a contact email address. A more detailed description of the ePOCS system, covering its technical components, data flows and involved human activities, can be found elsewhere [19].

### Developing and testing the ePOCS system within a context of hospital-based clinician-led patient recruitment

For any research involving PROMs collection, patients must be identified as eligible, informed about the study and provide consent. A system like ePOCS must, therefore, be able to receive information of newly recruited patients, and be developed with a view to the context(s) of recruitment. In the UK, it is not permitted to contact patients directly through cancer registries, and research suggests patient recruitment rates are generally higher in secondary than primary care [21-23]. Furthermore, we anticipate recruitment will be more effective and sustainable if undertaken by clinical teams than by non-NHS research personnel. In Yorkshire, where ePOCS is being developed and tested, the electronic patient record used in many hospitals is Patient Pathway Manager (PPM), which contains a research database in which all trials/studies are listed, all associated consented patients are 'tagged' as such, and all related study events are recorded (e.g. date of consent) [24]. The most efficient means of supplying the ePOCS system with notice and information about newly consented patients, from the hospital-based recruiting clinical teams, will be via this electronic record (i.e. via a daily automated data feed from PPM to the ePOCS system). Therefore, we have decided to develop and test ePOCS, at least in the first instance, within a context of secondary-care based patient recruitment, which is led by clinical teams (supported by research nurses), from hospitals using an electronic patient record (PPM).

### Study aims

#### Primary aim

The primary aim of this study is to test the feasibility of the ePOCS system, by running it for 2 years, in two Yorkshire NHS Trusts, with non-metastatic breast, colorectal and prostate cancer patients (largest survivor groups), and using the Northern and Yorkshire Cancer Registry and Information Service (NYCRIS). System feasibility will be examined primarily in terms of patient recruitment and retention rates, the representativeness of participating patients, the quantity and quality of the collected PROMs data, patients' feedback, the success and reliability of the underpinning informatics, and the system running costs.

#### Secondary aims

We also aim to analyse the PROMs data collected during system feasibility testing. If ePOCS proves feasible, and data of satisfactory quality are collected from a sufficient number of patients, we plan to examine these data for two main purposes: (1) to explore and check the psychometric properties (e.g. reliability, validity) of the newer, less well-established PROMs requiring of further psychometric analyses, and (2) to assess and describe patients' self-reported health and quality-of-life outcomes, and explore if and how they relate to disease, treatment and/or individual differences characteristics.

## Methods/Design

### Study funding and approvals

This study is funded by Macmillan Cancer Support, and is sponsored by the University of Leeds. The study protocol has received ethical and governance approvals from the Leeds East NHS Research Ethics Committee (ref. 10/H1306/65), and from the Leeds Teaching Hospitals NHS Trust and Calderdale and Huddersfield NHS Foundation Trust Research and Development Departments. Small amendments to the protocol, concerning the methods of recruitment and the PROMs used, have also subsequently been approved and are included in this paper.

### Study setting

Patients will be recruited from the Yorkshire Cancer Network (YCN), which covers a population of approximately 2.6 million in the North and West Yorkshire areas of England, UK. North and West Yorkshire include ethnically and socioeconomically diverse urban and rural areas. Patients will be recruited from five hospitals in the Leeds Teaching Hospitals NHS Trust, which is the YCN Cancer Centre, and from two hospitals in the Calderdale and Huddersfield NHS Foundation Trust, which is one of six YCN Cancer Units.

### Participants and sample size

Patients will be eligible for study inclusion if they are: (1) over 17 years of age, (2) diagnosed with breast, colorectal or prostate cancer, (3) within the last 6 months, (4) suitable for treatment with curative intent, and (5) English literate. Patients will be excluded if they lack the capacity to give informed consent (e.g. due to psychopathology, cognitive dysfunction, learning difficulties). The primary aim of the study is to test the feasibility of the ePOCS system, and one of the key feasibility outcomes is the number of patients who consent to join-up (as a proportion of all those eligible and invited). The recruited sample size is therefore an unknown outcome, rather than a predetermined target; we aim to approach all eligible consecutive patients and to recruit all those who are willing to consent. However, we have estimated that we may expect to recruit around 600 patients. The number of adults newly diagnosed with breast, colorectal or prostate cancer, within a recent 12 month period at the two participating NHS Trusts, is almost 4,000 (specifically 3,839). However, many patients will not meet the full eligibility criteria (e.g. some patients will be non-English literate, unable to provide informed consent etc.) (3,839 × .50 = 1,919), and we plan to recruit patients for approximately eight months, rather than a whole year (1,919 × .66 = 1,266). Previous similar PROMs-based studies run by our research group have yielded approximately 70% consent rates [25-28] (1,266 × .70 = 886), although not all patients will have sufficient interest in and/or access to the internet to consent to join ePOCS-most likely those without home access [29] (886 × .70 = 620).

### Patient Reported Outcome Measures (PROMs)

Patients will be asked to complete a range of generic, cancer-specific and cancer diagnosis-specific PROMS, representative of those likely to be administered in future research studies which use the ePOCS system. Patients will be asked to complete PROMs at three time-points; as soon as possible after consent (≤ 6 months post-diagnosis; T1), 9 months post-diagnosis (T2), and 15 months post-diagnosis (T3). The specific measures are detailed below, and assess a variety of health and quality-of-life domains, including pain, fatigue, psychological well-being, physical, social and sexual functioning and financial concerns.

#### Illness Perception Questionnaire-Revised (IPQ-R)

The IPQ-R [30] assesses patients' personal beliefs and expectations about their illness (e.g. about its controllability and consequences), and can be adapted to assess perceptions about any illness (i.e. in this instance, cancer). The IPQ-R comprises nine subscales, eight of which will be used in this study; the 'causes' subscale will be omitted due to concern from patients on our study steering group that enquiring about the (perceived) causes of a patient's cancer would cause distress; this use of subscales is valid (Moss-Morris, personal communication, 1 July 2010). The IPQ-R (minus the causes subscale) comprises 38 statements (e.g. "my cancer is a serious condition", "my cancer will improve in time") rated on a scale of 1 (*strongly disagree) *to 5 (*strongly agree)*, and 14 symptoms (e.g. 'breathlessness', 'headaches') rated on a *yes *(1)/*no *(0) scale, with respect to patients' views at the present moment. The IPQ-R, and its predecessor the IPQ [31], have been shown to be reliable and valid, and to predict various aspects of illness adaptation and recovery, in a range of samples including cancer patients [30,32-37]. Patients will be asked to complete the IPQ-R at T1.

#### EuroQol-5D (EQ-5D), version 2

The EQ-5D [38,39] is a 6-item generic measure of health status which assesses mobility, self-care, usual activities, pain/discomfort and anxiety/depression, using a three-option response format according to the severity of problems experienced that day (no problems, some problems, severe problems). The EQ-5D also includes a visual analogue scale on which *health state today *is rated from 0 (*worst imaginable health state*) to 100 (*best imaginable health state*). The EQ-5D is an internationally used measure, that can be employed in both the clinical and economic evaluation of health care, and which has been shown to be reliable and valid in several previous studies with cancer patients [40-43]. Patients will be asked to complete the EQ-5D at all three time-points.

#### Medical Outcomes Study 36-item Short-Form Health Survey, version 2 (SF-36v2)

The SF-36v2 [44] is a generic measure of health status and functioning which assesses eight domains including physical functioning, pain and mental health. The measure comprises 36 items (e.g. "have you been happy", "did you feel worn out") rated on a variety of Likert-type response scales (e.g. *excellent *- *poor*, *all of the time *- *none of the time*), primarily with respect to the past 4 weeks. The SF-36v2, and its predecessor the SF-36, are internationally used measures with extensive normative data, and have been demonstrated reliable and valid in numerous previous studies with cancer patients [45-50]. Patients will be asked to complete the SF-36v2 at T2 and T3.

#### European Organisation for Research and Treatment of Cancer (EORTC) Quality of Life Questionnaire (QLQ), version 3

The EORTC-QLQ [51] is a cancer-specific measure assessing health-related functioning and symptoms, which includes a generic core questionnaire and numerous diagnosis and treatment specific modules. This study will use the breast (EORTC-QLQ-BR23), colorectal (EORTC-QLQ-CR29) and prostate (EORTC-QLQ-PR25) modules, each of which contain between 20 and 27 questions (depending on item branching). In addition to the relevant diagnosis-specific module, patients will be asked to complete six subscales from the core questionnaire (nausea and vomiting, dyspnoea, insomnia, appetite loss, constipation and diarrhoea; 7 items), and two from the ovarian module (peripheral neuropathy and chemotherapy side effects; 7 items); this use of subscales is acceptable (EORTC, personal communication, 19 July 2010). All EORTC-QLQ items (e.g. "did you have a dry mouth", "has weight gain been a problem for you") are rated on a scale of 1 (*not at all*) to 4 (*very much*) with respect to the past week or month. The EORTC-QLQ is an internationally used measure with sound psychometric properties [5,25,51-54]. Patients will be asked to complete the EORTC-QLQ modules and subscales at T2 and T3.

#### Social Difficulties Inventory (SDI-21)

The SDI-21 [27] assesses various everyday difficulties commonly experienced by cancer patients, including relationship difficulties, domestic problems and financial worries; 21 questions (e.g. "have you felt isolated", "have you had any financial difficulties") are answered on a 0 (*no difficulty*) to 3 (*very much*) scale with respect to the past month. The SDI-21 has been demonstrated reliable and valid, has a clinically meaningful scoring system (including cut-off and minimal change scores indicative of need for action), and is highlighted in the NCSI as a potentially useful screening measure [1,27,28,55]. Patients will be asked to complete the SDI-21 at T2.

#### Quality of Life in Adult Cancer Survivors (QLACS) scale

The QLACS [56] measures health-related quality-of-life in seven generic and five cancer-specific domains, including cognitive problems, social avoidance and appearance. QLACS comprises 47 items (e.g. "you felt tired a lot", "you had difficulty doing activities that require concentrating") rated on a scale of 1 (*never*) to 7 (*always*) with respect to the past 4 weeks. QLACS is a relatively new measure, although is flagged as potentially useful in the NCSI, and evidence to date indicates acceptable psychometric properties in samples including breast, colorectal and prostate cancer patients [1,56-58]. Patients will be asked to complete the QLACS at T3.

#### Sociodemographic details (and clinical information)

Patients will be asked to provide information about their ethnicity, relationship status and level of education at T1. Other sociodemographic details (e.g. gender, age, postcode) and clinical information (e.g. date and type of cancer diagnosis, treatment regimens) will be obtained from participants' medical records (following their explicit permission, recorded on the consent form).

#### Employment status

Patients will be asked one question about their pre-diagnosis employment status at T1 (e.g. full-time employment, retired), and two questions about their current employment status at all three time-points (e.g. full-time employment, retired, currently working less or more hours than usual).

#### Past use of mental health services

Patients will be asked two questions about their past utilisation of mental health care services at T1; questions enquire about lifetime use, and use over the last 12 months, and originate from the National Comorbidity Survey [59-61].

#### Recent use of health and social services

Patients will be asked 20 questions about their use of health and social services at T3. The questions have been devised with health economist colleagues from the University of Leeds, and aim to help estimate some of the financial costs of cancer and its treatment, both to patients and the welfare state. Questions enquire about patients' frequency of use of medications and services (e.g. district nurses, social workers, residential homes, hospices), and the cancer-related costs incurred by patients and carers (e.g. due to increased home-heating costs, and travel and time off work for hospital appointments), over the last 3 months.

### Patient feedback on the ePOCS system

At the end of the study, participants will be offered the opportunity to provide comment on the ePOCS system through a 'feedback questionnaire' containing a mix of 28 closed and open questions (e.g. "what did you like about the electronic questionnaire system", "would you have preferred to complete the questionnaires on paper"). For patients who join the feasibility study and use the ePOCS system to complete PROMs, we want to know about their experiences, and whether they have any suggestions for the system's improvement. For patients who join the study but do not go online and complete (all the) PROMs, we would like to know if there were any system-related reasons for this, and if there are things we can change to make the system easier to use. It will be important to keep feedback on the system distinct from the system itself, and to include those patients who may join the study but do not in fact engage with the system and complete PROMs. Therefore, all consented patients (who have not actively withdrawn from the study) will be posted a paper copy of the feedback questionnaire, after completion of the T3 PROMs (or after the time-window for completion has expired). To minimise burden, a pre-paid addressed envelope will be included for return, and non-returns will not be followed-up with reminders.

### Procedures

#### Patient recruitment

Recruitment will be undertaken by oncology clinical care teams, at the two participating NHS Trusts, supported by dedicated research nurses funded by the West Yorkshire Comprehensive Local Research Network. Eligible patients will be identified during discussions in routine MDT meetings and/or through consultation of medical notes. All eligible patients will be given a comprehensive information sheet containing the contact details of the ePOCS study team, and which emphasises the voluntary nature of participation, and patients' right to withdraw consent, at any time, without the need for explanation, and without their personal care being affected. Patients who wish to join the study will be asked to read, complete and sign a consent form; this will be countersigned by the recruiting research nurse/clinician, and a copy filed in patients' medical notes. Participants' General Practitioners will be sent a letter informing them of their patients' participation in the study. Clinical care teams will provide anonymous information about those patients who decline to participate (gender, age, postcode, date and type of cancer diagnosis); if forthcoming, the recruiting teams will also note any reasons offered by patients for non-consent (e.g. lack of time, no interest in computers).

Prior to this study, we conducted a small opinion-gathering interview study (NHS Research Ethics Committee ref. 09/H1313/73) to obtain advice from oncology clinicians and patients about the 'best' time and person in secondary care to approach patients about joining ongoing PROMs-based research. Following the strong unanimous views expressed in that study, patients will first be approached about participation in the current feasibility study by an oncology clinician with whom they are familiar (e.g. consultant, registrar, specialist nurse) and, if possible, with whom they have established a 'good relationship'. Wherever possible, patients will be approached about the study in-person, typically during a routine outpatient appointment; if this is not possible, patients will be sent a letter about the study, signed by their consultant. Perhaps unsurprisingly, given that every patient is an individual, and every care pathway is unique, opinion in the interview study did not converge on a single best time for approach. Clinicians and patients did, however, agree on what were non-optimal times; notably, very close to the time of diagnosis (i.e. within a few weeks), and when patients are facing uncertainty (e.g. when the treatment course is yet to be decided, when the results of a short-term intervention like surgery are as yet unknown). The general consensus was that patients several weeks out from diagnosis, who are well-embarked on a decided course of treatment, will be open to receiving information about the study. However, the exact time-point when this will be (e.g. 4 weeks or 3 months post-diagnosis) will differ for each patient. The clinical teams recruiting patients will use the findings from our preparatory interview study to inform and guide their recruitment approach.

#### Patient participation

Consenting patients will be given a unique ID and password on a postcard, which contains instructions for accessing the ePOCS website and our study team's contact details. Patients will be asked to log-on to the ePOCS system and complete the T1 PROMs as soon as possible. Patients will then be sent an email or letter (depending on their preferred mode of correspondence) when they are approaching (i.e. 3 weeks before) 9 months (T2) and 15 months (T3) post-diagnosis, inviting them to log-on to the system and complete PROMs again. At each time-point, patients will have up to 6 weeks to complete the PROMs (i.e. 3 weeks either side of the exact time-point; aside from the first time-point, which is variable, as patients will be recruited at any time within 6 months post-diagnosis). Up to two email/letter reminders will be sent to patients who do not complete the PROMs. For all PROMs questions, patients will have the option to respond 'I would prefer not to answer this question'. Completion of the PROMs at each time-point is likely to take approximately 20-30 minutes in total; patients can spread-out completion if they wish (e.g. over a number of days). All correspondence will remind patients that their PROMs responses are not fed back to their clinical team, and to contact their doctor or nurse if they have any problems or concerns about their health. The study website also contains links to relevant supportive organisations (e.g. Macmillan Cancer Support, Prostate Cancer Charity). At the end of the study, patients will be sent a paper feedback questionnaire inviting their thoughts and opinions on the ePOCS system.

#### System administration

Members of our study team will administrate the ePOCS system during feasibility testing. We will use the Tracker on a daily basis to monitor patients' PROMs completion progress, and to prepare and send the necessary related communications (e.g. reminder notices, thank you acknowledgments). Our study team will also be accessible to patients (via email, telephone and/or letter) to provide assistance with using the ePOCS system.

The current feasibility study is funded for 2 years. If the ePOCS system proves feasible, we would like to expand the research by, amongst other things, following patients for longer than 15 months post-diagnosis. If we obtain further funding to do this, we would like to invite the patients who participate in this feasibility study to join our future studies (and complete more PROMs at longer-term follow-ups). We will explicitly seek patients' permission to contact them in the future (for up to 10 years after the study) if we obtain further funding-to inform them about, and to seek their consent to participate in, new ePOCS-related research studies. Patients will be informed of this via the study information sheet, and if they give permission for future contact, will be asked to sign a separate statement on the consent form to indicate this.

### Study outcomes and data analysis

#### Primary outcomes

The feasibility of the ePOCS system will be assessed by examining a number of different outcomes. Notably: (1) the proportion of patients recruited into the system, and their representativeness, relative to all those invited to join, (2) the proportion and representativeness of patients who remain in the system and complete PROMs at the different time-points, relative to all those who join, (3) the completeness, timeliness and reliability of the PROMs data obtained at each time-point (e.g. extent of missing responses, internal reliability of responses), (4) patients' views and opinions on the system provided via the feedback questionnaire, (5) the success and reliability of the underpinning informatics (e.g. frequency and nature of technical problems, frequency and nature of patient enquiries requesting assistance in using the system), and (6) the system running costs. The efficiency and practicality of the patient recruitment procedures (i.e. hospital-based clinician-led recruitment) will also be examined.

The different feasibility outcomes will be primarily analysed quantitatively using descriptive statistics such as proportions, frequencies and means (e.g. proportion of invited patients who consent, frequency of patient requests for help with using the system, mean running costs per month), chi-square and t-tests (e.g. to examine group differences in gender, age etc. between consenters and non-consenters) and measures of internal reliability (e.g. Cronbach's alpha, to assess quality of PROMs responses). Open-ended patient feedback questions will be examined qualitatively, as will the type of reasons offered for non-consent and withdrawal, and the nature of technical problems and patient difficulties with using the system. The efficiency and practicality of the patient recruitment procedures will also be examined qualitatively (including using process mapping tools and software).

#### Secondary outcomes

If the system proves feasible, and a sufficient number of patients provide data of satisfactory quality, we will analyse these data for two main purposes: (1) to explore and check the psychometric properties (e.g. reliability, validity) of the newer, less well-established PROMs requiring of further psychometric analyses, and (2) to assess and describe patients' self-reported health and quality-of-life outcomes, and explore if and how they relate to disease, treatment and/or individual differences characteristics.

Psychometric analyses will be undertaken using descriptive statistics such as proportions and means (e.g. to determine the presence of floor and ceiling effects), measures of correlation (e.g. between responses to different PROMs to determine convergent and discriminant validity) and measures of internal reliability (e.g. Cronbach's alpha). The health and quality-of-life outcomes reported by patients will be assessed and described using statistics such as proportions, means and standard deviations (e.g. sample means and standard deviations of PROMs scores) and t-tests (e.g. to compare the sample scores with population norms). If and how the outcomes relate to disease, treatment and/or individual differences characteristics (e.g. sociodemographic and psychological variables) will be explored using multiple and logistic regression analyses (e.g. to explore if illness perceptions within 6 months of diagnosis are predictive of health outcomes 15 months post-diagnosis) and/or analysis of variance (ANOVA) (e.g. to examine if breast, colorectal and prostate cancer patients experience significantly different levels of social difficulties 9 months post-diagnosis).

### Study organisation and management

The study research team and wider steering group collectively includes expertise in psychosocial oncology research, (electronic) PROMs, clinical oncology, nursing, data management, statistics, epidemiology, health informatics and cancer registration. The conduct and progress of the study will be discussed and reviewed in fortnightly to monthly meetings of the core day-to-day research team and chief investigator, and in quarterly meetings of all study co-investigators/the wider steering group. The steering group includes four patient representatives, and we will provide verbal reports on the study at biannual meetings of our wider research group's patient and carer Research Advisory Group. The study is included in the UK National Institute for Health Research (NIHR) Clinical Research Network (CRN) Portfolio, and we will provide monthly anonymised reports on study accrual to the NIHR CRN office. We will also provide regular reports on study recruitment and progress to the National Cancer Research Institute clinical studies groups (the breast, colorectal and prostate cancer site-specific groups, and the psychosocial oncology group).

## Discussion

### System usability for patients

Most cancer survivors are older (> 65 years) [10], with associated lower levels of computer literacy [62]. However, we have borne this in mind during the development of the ePOCS system, and worked with cancer patients to include features which will enhance usability for older adults and/or those with limited computer skills. For example, all website and QTool webpages have large-size text and navigation buttons, and QTool saves answers to PROMs entirely automatically; a detailed account of the system features included to increase patient usability is provided elsewhere [19]. We acknowledge that not all patients will be sufficiently interested or enabled to use an internet-based system like ePOCS. However, ePOCS is a future-focused system, and the 'digital divide' is a diminishing problem. In the UK, the number of internet-enabled households and individuals is continually increasing; in 2010 73% of households had internet access, compared with only 57% in 2006, and 73% of adults used the internet at least weekly, relative to just 51% in 2006 [29]. However, it is likely inevitable that, for the short-term future, paper PROMs will have to be made available as a 'back-up' option for a (ever-decreasing) proportion of patients.

### The potential benefits and uses of the ePOCS system

This study aims to test the feasibility of an electronic system in which PROMs are completed online and the data are stored in cancer registries/the NCDR. As the Internet and cancer registries both provide enduring UK-wide coverage, such a system is feasibly sustainable and UK-scalable. ePOCS has potential to provide a cost-efficient technical platform to facilitate and support longitudinal cohort research to improve understanding of cancer survivors' experiences. Improved understanding of the challenges of survivorship will enable cancer patients (if they wish) to receive more detailed, individualised information about the symptoms and psychosocial difficulties they may face ahead, based on the self-reported experiences of other similar patients. It will also inform service planning and facilitate the development of tailored interventions for those patients who (or who will likely) experience significant difficulties. The ePOCS system could potentially be used by different groups for different purposes. The system could be used by university research groups, independently but simultaneously, to facilitate and support different PROMs-based survivorship cohort studies. Alternatively, the system could be used in a national government-supported initiative to realise the NCSI goal of increased cancer PROMs collection; if ePOCS is used in this way, the resultant anonymised dataset could be made a national resource, freely available on request for hypothesis-driven analyses.

## Competing interests

The authors declare that they have no competing interests.

## Authors' contributions

DF, AN, JB, AD, GV and PW conceptualised the ePOCS project, created the original protocol, and obtained study funding; PW is the chief investigator, and DF, AN, JB, AD and GV are co-investigators. PW, LA and HJ led and managed the design and build of the ePOCS system, and developed the design and methods for the current feasibility test study. LA drafted and prepared the study protocol and the current manuscript (which has been read and approved by all authors). HJ and LA oversee the day-to-day running of the study.

## Pre-publication history

The pre-publication history for this paper can be accessed here:

http://www.biomedcentral.com/1472-6947/11/66/prepub
